# Comparison of the Efficacy of Metformin and Lifestyle Modification for the Primary Prevention of Type 2 Diabetes: A Meta-Analysis of Randomized Controlled Trials

**DOI:** 10.7759/cureus.47105

**Published:** 2023-10-16

**Authors:** Jaahnavi Vajje, Saima Khan, Avneet Kaur, Heemali Kataria, Sahar Sarpoolaki, Aastha Goudel, Ali Hanif Bhatti, Danish Allahwala

**Affiliations:** 1 Internal Medicine, Dr. Pinnamaneni Siddhartha Institute of Medical Sciences and Research Foundation, Vijayawada, IND; 2 Internal Medicine, Sir Syed College of Medical Sciences for Girls, Waterbury, USA; 3 Internal Medicine, Government Medical College, Patiala, Patiala, IND; 4 Internal Medicine, Government Medical College, Surat, Surat, IND; 5 Medicine, Universidad Interamericana de Panamá, Panama, PAN; 6 Medicine, Zainul Haque Sikder Women's Medical College and Hospital, Dhaka, NPL; 7 Internal Medicine, Lawrence General Hospital, Lawrence, USA; 8 Nephrology, Fatima Memorial Hospital, Karachi, PAK

**Keywords:** meta-analysis, lifestyle intervention, prevention, type 2 diabetes, metformin

## Abstract

This meta-analysis aimed to compare the effectiveness of metformin versus lifestyle interventions in preventing diabetes in individuals with prediabetes. We followed the Preferred Reporting Items for Systematic Reviews and Meta-Analyses (PRISMA) guidelines and conducted a comprehensive search of databases (PubMed, Cochrane Library, EMBASE) up to September 1, 2023. Five eligible studies were included. The results showed that there was no significant difference in the risk of developing diabetes between the metformin and lifestyle intervention groups (RR: 1.14, 95% CI: 0.77-1.68). Similarly, when comparing metformin with lifestyle intervention, the risk of diabetes was slightly higher in the metformin group, but this difference was not statistically significant (RR: 1.31, 95% CI: 0.93-1.86). When comparing metformin with lifestyle intervention to lifestyle intervention alone, no significant difference was observed in the incidence of diabetes (RR: 0.88, 95% CI: 0.74-1.04). In conclusion, our analysis found that the incidence of type 2 diabetes was slightly higher in patients receiving metformin alone compared to lifestyle intervention, but this difference did not reach statistical significance. Further trials are necessary to better evaluate these interventions for preventing type 2 diabetes in high-risk individuals.

## Introduction and background

Type 2 diabetes mellitus presents a significant global health challenge characterized by a considerable burden of morbidity and mortality, affecting roughly 5% of adults worldwide, and its prevalence is on a steep upward trajectory [1]. Projections indicate that this number will escalate to 578 million individuals by 2030 and may reach 700 million by 2045, which would account for approximately 10.9% of the global population [2]. Diabetes is associated with a substantial array of complications, including heart disease, stroke, neuropathy, nephropathy, and retinopathy [3]. The development of type 2 diabetes is attributed to a complex interplay of genetic, environmental, and behavioral factors, including a sedentary lifestyle and a diet high in calories but deficient in essential nutrients, both contributing to the risk of obesity [4]. This escalating incidence and prevalence of diabetes is a concern shared by both developed and developing nations, with the added concern of a decreasing age of onset [5]. Furthermore, the chronic nature of diabetes and its associated consequences exacerbate the suffering of patients and their families while also imposing significant costs on healthcare systems and society at large [2].

Individuals with prediabetes face an elevated risk of progressing to full-blown diabetes, offering an opportunity for early detection and intervention to prevent new cases of diabetes [6]. According to guidelines from the American Diabetes Association, individuals with prediabetes are advised to adopt lifestyle modifications, including healthier dietary choices, increased physical activity, and weight management, to thwart the onset of diabetes [7]. Additionally, there is consideration of prescription medications for the prediabetic population. Scientific evidence supports the use of not only traditional antidiabetic medications such as metformin and acarbose but also newer agents such as GLP-1 receptor agonists in averting the development of diabetes. Several randomized controlled trials (RCTs) have explored various strategies for diabetes prevention, including both lifestyle modifications and pharmacological interventions [8-9].

Metformin is indeed a commonly prescribed medication for individuals with prediabetes and type 2 diabetes. It belongs to a class of drugs known as biguanides and is often used as a first-line treatment for managing elevated blood sugar levels [10]. Metformin primarily works by improving the sensitivity of your body's cells to insulin, which is the hormone responsible for regulating blood sugar levels. In prediabetes and type 2 diabetes, the body's cells become less responsive to insulin, leading to elevated blood sugar levels. Metformin helps the cells absorb glucose more effectively, reducing the amount of sugar in the bloodstream [11-12].

This meta-analysis aims to assess the comparative efficacy of metformin versus lifestyle interventions in preventing diabetes among individuals with prediabetes. While previous studies have explored these approaches individually, this analysis seeks to provide a more comprehensive evaluation, particularly in cases where sample sizes in prior research may have been limited.

## Review

Methodology

The current meta-analysis was conducted and reported following the Preferred Reporting Items for Systematic Reviews and Meta-Analyses (PRISMA) statement.

Search Strategy

Two reviewers independently searched online databases, including PubMed, Cochrane Library, and EMBASE, from the inception of databases up to September 1, 2023, without limitations on country or language. The search utilized the following key terms: "metformin," "type 2 diabetes," "prevention," and "lifestyle," along with their synonyms and medical subject heading (Mesh) terms. The search strategy was adapted to meet the requirements of each database. Additionally, the reference lists of all included studies were manually screened to identify any additional studies relevant to the study objective.

Study Selection

Articles were included in this meta-analysis if they compared metformin and lifestyle interventions in individuals aged 18 years or older. Studies reporting the required outcomes were considered. Observational studies, reviews, meta-analyses, and editorials were excluded from this meta-analysis. All studies obtained from online database searches were imported into EndNote X9. After removing duplicates, initial screening was performed based on abstracts and titles. Full-text assessment of eligible records was conducted, applying pre-defined inclusion and exclusion criteria. Two authors independently screened the articles, and any disagreements between them were resolved through discussion.

Data Extraction and Outcomes

Two independent reviewers conducted data extraction using a pre-designed data extraction form. Any discrepancies in the extracted data were resolved through discussion and consensus. The extracted information included study details, baseline characteristics of subjects, intervention details, and outcomes related to the risk of diabetes.

Risk of Bias Assessment

The methodological quality of the included RCTs was evaluated using the Cochrane Collaboration's Risk of Bias Tool within the review manager. This assessment covered aspects such as random sequence generation, allocation concealment, blinding of participants and personnel, blinding of outcome assessors, handling of incomplete outcome data, selective outcome reporting, and other potential biases. Each study was categorized as having a high, unclear, or low risk of bias. Two authors conducted the quality assessment, and input from a third reviewer was sought to resolve any disagreements.

Data Analysis

Data analysis was carried out using RevMan version 5.4.1 (the Cochrane Collaboration). The risk of diabetes was compared between the two groups using risk ratios (RR) with a 95% confidence interval (CI). Heterogeneity among the study results was assessed using I-squared. I-squared values exceeding 50% indicate significant heterogeneity. Subgroup analysis was performed, comparing the risk of diabetes in the metformin group and the metformin plus lifestyle intervention group separately. The significance level was set at a p-value of 0.05.

Results

**Figure 1:**
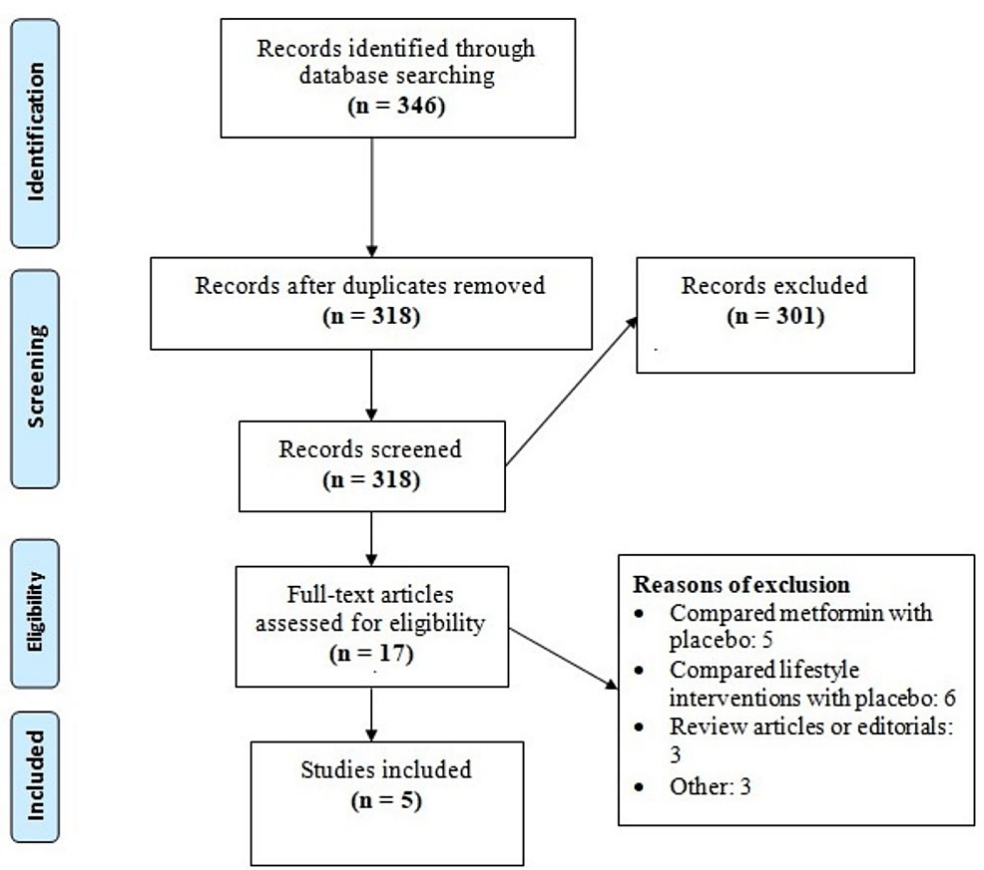
PRISMA flowchart of the study selection

**Figure 2:**
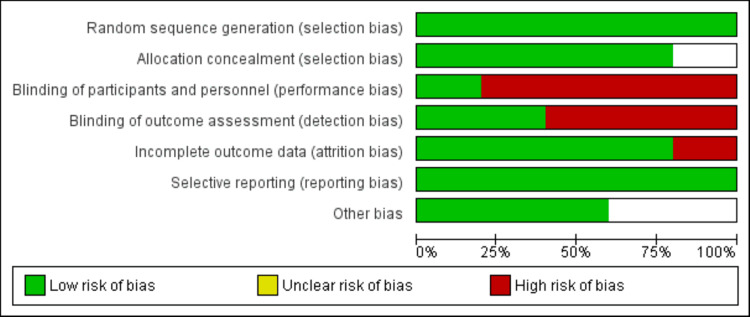
Risk of the bias graph for the included studies

We found 346 studies through online database searching. After the removal of duplicates, initial screening was done using abstracts and titles. Full text of 17 studies was obtained, and a detailed assessment was done based on pre-defined inclusion and exclusion criteria. Finally, five studies met the eligibility criteria and were included in this meta-analysis. Figure 1 shows the process of study selection. Table 1 shows the characteristics of the included studies. Table 2 shows the description of lifestyle intervention used by each of the included studies. Figure 2 presents a risk of bias assessment of all included studies.

**Table 1:** Characteristics of the included studies NR: Not reported

Author Name	Year	Setting	Groups	Sample Size	Follow-up	Mean Age (years)	Males (n)	Family history of diabetes (n)
Knowler et al. [13]	2002	Multicenter	Metformin	1073	2.8 years	50.9	363	733
			Lifestyle intervention	1079		50.6	345	752
Lu et al. [14]	2011	Single center	Metformin	95	2 years	62.44	45	NR
			Lifestyle intervention	86		64.72	50	NR
O’Brien et al. [15]	2017	Single center	Metformin	27	1 year	45.8	NR	21
			Lifestyle intervention	30		45.5	NR	25
Ramachandran et al. [16]	2006	Single center	Metformin	133	3 years	45.9	104	55
			Lifestyle intervention	133		46.1	107	68
Zhang et al. [17]	2023	Multicenter	Metformin	831	2 years	NR	NR	NR
			Lifestyle intervention	847		NR	NR	NR

**Table 2:** Lifestyle intervention incorporated by the included studies

Study ID	Lifestyle Intervention	Metformin
Knowler et al. [13]	The lifestyle modification program consisted of 16 one-on-one counseling sessions spanning 24 weeks. These sessions covered topics related to diet, physical activity, and behavior adjustment. The objectives were to achieve a 7% reduction in body weight and engage in at least 150 minutes of weekly physical activity. The maintenance phase involved ongoing individual sessions, typically held on a monthly basis, and group sessions, which were part of the Diabetes Prevention Program.	850 mg twice daily
Lu et al. [14]	Participants in the intervention group received face-to-face lifestyle coaching sessions every quarter and had monthly phone consultations.	250 mg thrice daily
O’Brien et al. [15]	The intensive lifestyle interventions (ILI) were based on the Diabetes Prevention Program's ILI and were delivered by community health workers, referred to as promoters, over the course of 24 sessions.	850 mg twice daily
Ramachandran et al. [16]	The lifestyle modification group was motivated to engage in 30 minutes of daily exercise and enhance their dietary habits through monthly phone conversations and in-person meetings every six months.	250 mg twice daily
Zhang et al. [17]	Intervention included dietary teaching with physical exercise.	850 mg twice daily

Risk of Diabetes

**Figure 3:**
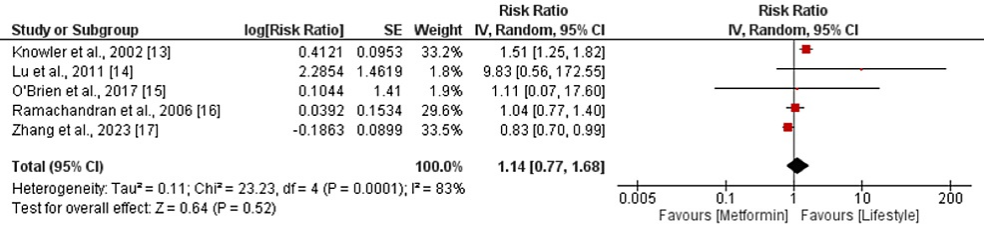
Comparison of the risk of diabetes between the two groups References [13-17]

A pooled analysis of five studies showed that the risk of developing diabetes was not significantly different between patients who were in the metformin group and patients in the lifestyle intervention group (RR: 1.14, 95% CI: 0.77-1.68), as shown in Figure 3. We performed subgroup analysis on the basis of co-intervention in the metformin group. The risk of diabetes was higher in patients in the metformin group compared to those patients in lifestyle intervention. However, the difference was statistically insignificant (RR: 1.31, 95% CI: 0.93-1.86). In the other group, where a comparison was made between metformin with lifestyle intervention and lifestyle intervention alone, no significant difference was there between the two groups in terms of incidence of diabetes (RR: 0.88, 95% CI: 0.74-1.04).

Discussion

This study was conducted to assess the efficacy of metformin in reducing the incidence of type 2 diabetes in patients at high risk of diabetes. The study concluded that the incidence of type 2 diabetes was higher in patients receiving metformin alone compared to those with lifestyle intervention.

Previous studies have reported that both lifestyle modification and drug therapy are effective in preventing diabetes compared to the control group. For instance, pooled results from 31 trials with 4,570 participants followed by 8,300 patients-years found that metformin treatment reduced the risk of diabetes compared to the control group [18]. The meta-analysis conducted by Schellenberg et al. reported that lifestyle interventions efficiently reduce the incidence of type 2 diabetes in high-risk patients [19]. It is reported in the present meta-analysis that the combination of lifestyle interventions and metformin is more effective in reducing the risk of diabetes and has a synergistic effect. However, only two studies, The RCT conducted by Orchard et al. reported that, compared to metformin, lifestyle interventions are more effective in reducing metabolic syndromes [20]. Another study found that lifestyle interventions were more effective than metformin in preventing diabetes in women with gestational diabetes. The study found that the lifestyle intervention group had a 50% reduction in the risk of diabetes, compared to a 31% reduction in the metformin group [21].

In general, interventions targeting individuals at risk have proven effective in reducing the likelihood of developing type 2 diabetes. Specifically, when examining two key risk factors associated with diabetes, namely, obesity and reduced physical activity, lifestyle interventions designed to address these factors have demonstrated greater efficacy in reducing diabetes incidence. Interestingly, it appears that the impact of lifestyle interventions is more pronounced in individuals with higher body mass index (BMI). While pharmaceutical interventions have also shown promise in reducing diabetes risk, these effects tend to diminish after the cessation of treatment. For instance, discontinuing the use of two drugs, troglitazone and metformin, led to an increase in diabetes incidence [22]. While our meta-analysis did not directly assess the effectiveness of lifestyle modification and metformin interventions, our final results align with and corroborate these findings.

Furthermore, a network meta-analysis (NMA) study indicates that lifestyle modification interventions outperform 12 other therapeutic approaches for preventing type 2 diabetes in high-risk populations. However, when indirectly comparing lifestyle modification and metformin, the odds ratio for diabetes risk reduction showed a 14% decline, though this difference did not reach statistical significance (95% CI: 1.25-0.6, OR: 86%) [23]. It is worth noting that this inconsistency with our study is likely attributable to the smaller number of studies included in our meta-analysis.

One of the disadvantages of lifestyle interventions is that it is more challenging to adhere to. While lifestyle intervention remains the fundamental approach for preventing diabetes, its effectiveness is constrained by the challenge of maintaining adherence and sustaining behavioral changes over the long term. This limitation results in less-than-ideal glycemic control and diminishes the protective benefits over extended periods [24]. Therefore, the best approach for preventing diabetes in high-risk individuals may be to combine medication and lifestyle interventions [24]. Lifestyle interventions demand more active participation and effort but can provide a more comprehensive approach to diabetes prevention. Adherence to lifestyle changes may vary widely among individuals, but when successfully implemented and maintained, they offer the potential for long-term health benefits beyond diabetes prevention [25].

Optimal approaches to identifying candidates for preventive measures remain to be determined, and only two studies compared the combination of metformin and lifestyle interventions with intervention alone. Therefore, future studies need to focus on this particular group to address the increasing incidence of diabetes. Secondly, subgroup analysis needs to be done to predict whether the effect of different interventions is similar in persons with an isolated elevation of the fasting or post-load glucose concentration or individuals with other risk factors for diabetes.

Study Limitations

This meta-analysis has specific limitations that need to be acknowledged. Firstly, the inclusion of a relatively small number of studies in this analysis is a constraint. Secondly, the issue of adherence represents a significant challenge in interventions aimed at preventing diabetes, yet a majority of the studies included in our analysis did not evaluate adherence. As a result, we were unable to assess adherence in this meta-analysis. Hence, it is imperative for forthcoming research endeavors to incorporate assessments of adherence. This will allow for a more comprehensive evaluation of the compatibility and effectiveness of these interventions.

## Conclusions

Our investigation has brought to light a critical finding: individuals who rely solely on metformin as a preventive measure exhibit a higher susceptibility to developing type 2 diabetes when compared to those who engage in lifestyle interventions, but the difference was not statistically significant. This noteworthy discovery underscores the multifaceted nature of diabetes prevention, where pharmacological intervention alone may not suffice to provide the desired level of protection. However, the narrative takes an intriguing turn when we consider the combined approach of metformin and lifestyle interventions. The synergy observed in this combination therapy is striking. It becomes evident that the joint action of metformin and lifestyle modifications provides a more robust defense against the onset of type 2 diabetes. To further advance our understanding of preventive measures, future studies should focus on the specific group that combines metformin and lifestyle interventions, addressing the rising incidence of diabetes.
